# Household Transmission of SARS-CoV-2 in Bhutan

**DOI:** 10.1155/2022/5644454

**Published:** 2022-07-08

**Authors:** Jimba Jatsho, Dorji Pelzom, Sithar Dorji, Thinley Pelzang

**Affiliations:** ^1^Department of Pediatrics, Phuentsholing Hospital, Chukha, Bhutan; ^2^Policy and Planning Division, Ministry of Health, Bhutan; ^3^Phuentsholing Hospital, Chukha, Bhutan

## Abstract

**Introduction:**

The transmission trend of SARS-CoV-2 is continuously evolving. Understanding the dynamics in different settings is crucial for any effective containment measures. We aimed to study the characteristics of household transmission of SARS-CoV-2 in Bhutanese households by determining the transmissibility within household contacts of confirmed COVID-19 index cases and their factors of infectivity.

**Methods:**

We conducted a retrospective observational study on household transmission in 306 household contacts of 93 COVID-19 positive index cases diagnosed from April 16, 2021, to June 30, 2021. A pro forma was used to collect data on the epidemiological, demographic, and clinical profile of all recruited individuals. Secondary attack rates (SAR) were calculated, and risk factors for transmission were estimated.

**Results:**

180 of 306 household contacts developed secondary household transmission (SAR 58.8%; 95% CI: 53.2-64.2). The median age of household contacts was 22 years. The median household size was 4 (mean 4.3 ± 2.199) members. Contacts exposed to adult index cases (aPR 1; 95% CI 1, 1.02, *p* = 0.01) and vaccinated index cases (uPR 0.41, 95% CI 0.25, 0.66, *p* < 0.001) had a higher SAR and prevalence of secondary infections.

**Conclusions:**

Our findings suggest substantial evidence of secondary infections among household contacts, especially in the context of public health mandated lockdowns. Aggressive early contact tracing and case identification with subsequent case isolation from other household members remains a crucial step in preventing secondary transmission.

## 1. Introduction

A novel coronavirus that started as an outbreak of atypical pneumonia in Wuhan, China, quickly spread, resulting in a global pandemic. The virus was named severe acute respiratory syndrome coronavirus 2 (SARS-CoV-2), and the disease associated with it is referred to as COVID-19 [1]. Recent figures from the World Health Organization (WHO) reveals 523,786,368 confirmed cases of COVID-19 and 6,279,667 deaths globally [2]. Bhutan has recorded 59,614 confirmed COVID-19 cases with 21 deaths as of 23 May 2022 [3].

The current evidences in COVID-19 transmission dynamics remain complex and are influenced by numerous interrelated factors. In order to achieve effective control of the pandemic, understanding the transmission dynamics of the virus is crucial. Setting or population specific transmission will facilitate identification of drivers of the epidemic and help direct public health control measures for control and prevention of outbreaks [4, 5].

Household transmission has become an important part of SARS-CoV-2 transmission, with studies showing household contacts of infected cases as the highest risk exposure setting [6, 7]. The WHO-China Joint Mission reported that human-to-human transmission cases were mostly clustered in family households [8]. It is also thought that the ongoing increase in cases even after national lockdowns and circuit breakers coupled with social-distancing rules imposed by countries may have been contributed by household transmission [9].

Transmission dynamics are also invariably affected by the emergence of new variants and increasing vaccination coverage in the population. Recent evidences suggest that Delta and Alpha variants have significantly higher secondary attack rates (SARs) than the original wild-type variant. Additionally, SARs in households with fully vaccinated index cases were lower than those with unvaccinated index cases. Transmission was also lower in fully and partially vaccinated than unvaccinated household contacts [10].

An updated living systematic review estimated an overall household SAR of 18.9% with transmission rates widely ranging from 3 to 74% [11]. However, published data on household SAR in the neighboring Southeast Asian countries are limited to few studies from India, Bangladesh, and Singapore in the last two years [12–15].

In Bhutan, household-specific transmission has not been studied yet. This study is aimed at describing the characteristics of household transmission of SARS-CoV-2 by estimating the SAR and determining the factors for infectivity in household contacts.

## 2. Materials and Methods

### 2.1. Study Design

This was a retrospective observational study of the data records maintained with the surveillance and contact tracing unit of Phuentsholing Hospital, Bhutan. Demographic and case investigation data from individual index cases and their corresponding household contacts were identified, and their data were collected.

### 2.2. Study Setting and Duration

Phuentsholing is a high risk area as it shares border with India and is the main gateway for all commercial activities of import and export for the country. Lockdown measures were imposed for the second time in April 2021 after community cases were detected. The study duration was from April 16 to June 30, 2021. The last date for contact follow-up was July 21, 2021.

### 2.3. Classification of Index Cases and Household Contacts

An index case was defined as the first case of laboratory-confirmed COVID-19 case detected on nasopharyngeal RT-PCR test in a family or household. All index cases and closed contacts living in the same household with no other identified sources of transmission apart from the index case were included.

A household contact was defined as any person, inclusive of close family members, neighbors, friends, and employers/employees, who are living in the same household unit as a confirmed COVID-19 index case. Close contacts from settings other than household, index cases with no close household contacts (living alone), and all imported cases detected from incoming travelers in 21 days mandatory facility quarantine were excluded from the study. As default, the first case detected from a household was considered as the primary index case based on laboratory confirmation and symptom onset. In cases of more than one case detected from a household, the onset of symptoms was considered to retrospectively identify the index case.

All household contacts of the index case were identified and screened with nasopharyngeal RT-PCR tests and were quarantined for 21 days, regardless of symptoms. All primary close contacts were followed up under quarantine for 21 days with regular nasopharyngeal RT-PCR tests at 3, 7, 14, and 21 days. Daily clinical follow-up was conducted via telephone for the full duration of the quarantine period. Contacts were tested in between the testing days whenever they became symptomatic.

Household contacts with children and parents were usually quarantined together in the same room for convenience and care. In certain unavoidable circumstances where the confirmed index case was a child who needed direct care by an uninfected guardian, the parent/guardian were provided with appropriate personal protective gear like N95 mask and gowns.

### 2.4. Variables

Epidemiological and demographic characteristics including sex, age, setting of detection, symptom status, high-risk group, size of household, vaccination status, relationship to index cases, and number of household contacts were collected.

Variables for risk factor (independent variables) analysis included age, sex, size of household, symptoms status, relationship to index case, and vaccination status.

Symptom data was collected at the time of positive PCR detection, during the positive case investigation. The variable of symptomatic or asymptomatic pertains to symptom status at the time of the positive PCR test.

### 2.5. Variable Definitions

Setting of detection was defined as places from where the index cases were detected. Any occupation directly dealing with suspected/positive cases with a high COVID-19 exposure risk were defined as “high risk group.” This included the police, immigration/customs, quarantine workers, essential delivery workers, health workers, and essential drivers. Symptomatic case was defined as a COVID-19 positive case with presence of symptoms suggestive of COVID-19 like fever, cough, fatigue, anorexia, shortness of breath, myalgia, sore throat, nasal congestion, headache, diarrhoea, nausea and vomiting, and loss of taste and smell. Asymptomatic case was defined as a COVID-19-positive case without any clinical symptoms of COVID-19. Mild symptoms are defined as symptomatic patients meeting the case definition for COVID-19 without evidence of viral pneumonia or hypoxia. Moderate symptoms are defined as symptomatic patients meeting case definition for COVID-19 with clinical signs of nonsevere pneumonia. Severe symptoms were defined as symptomatic patients meeting case definition for COVID-19 with clinical signs of severe pneumonia. Severe pneumonia in adults is defined as signs of pneumonia (fever, cough, and dyspnea) plus one of the following: respiratory rate > 30 breaths/min; severe respiratory distress; or SpO2 < 90% on room air. Severe pneumonia in children is defined as signs of pneumonia (cough or difficulty in breathing and fast breathing or chest wall indrawing) plus at least one of the following: SpO2 < 90%, very severe chest wall indrawing, grunting, central cyanosis, or presence of any other general danger sign (inability to breastfeed or drink, lethargy or unconsciousness, or convulsions).

Comorbidity was defined as having any of the following conditions: heart diseases, congenital heart disease, myocardial infarction, hypertension, diabetes, asthma, epilepsy, seizures, dementia, stroke, kidney disease/failure, pregnancy, cancer, peptic ulcer, or any other significant medical conditions. A person was considered to be vaccinated if he/she had received at least one dose of COVID-19 vaccine.

### 2.6. Data Collection

All reported COVID-19 cases from April 16 to June 30, 2021, were collected to identify index cases and their eligible household contacts. All contacts of index cases till June 30, 2021, were followed up till the end of their quarantine period of 21 days. Data was extracted from the surveillance, contact tracing, and medical records using a structured pro forma.

### 2.7. Statistical Analysis

Data was double entered and validated using EpiData (version 3.1, EpiData Association, Odense, Denmark), and data analysis was performed using STATA 13.1. Descriptive statistics for the index case and household contacts has been presented; continuous variables are expressed as mean/median and standard deviation/IQR as appropriate, and categorical variables are expressed as frequencies and proportions. SARs have been presented as percentages. Household SARs were estimated by dividing the total positive household contacts by the total household contacts exposed excluding the index case. Prevalence ratios with 95% confidence intervals were used to calculate the factors for infectivity among positive and negative contacts. A generalized linear model for binomial family was used to calculate the *p* value. Variables with *p* value ≤ 0.2 from unadjusted regression analysis were taken into consideration for further adjusted prevalence ratio.

### 2.8. Ethical Approval

Ethical approval was granted by the Research and Ethics Board of Health (REBH), Thimphu vide approval number REBH/Approval/2021/080 dated June 25, 2021. Informed consent for participants was waived by the ethics board. Administrative clearance was accorded by the Ministry of Health and administration of Phuentsholing Hospital.

## 3. Results

### 3.1. Study Population

**Figure 1:**
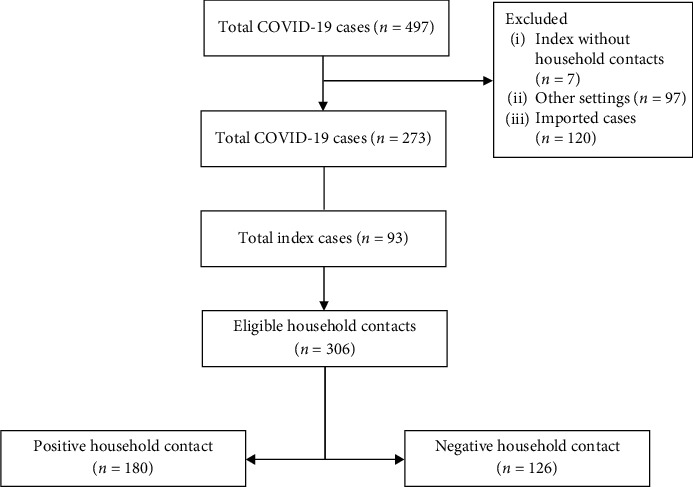
Flow algorithm of household cases.

During the study period from April 16 to June 30, 2021, a total of 497 laboratory-confirmed COVID-19 cases were detected. Among them, 93 index cases and their 306 eligible household contacts which fit the study inclusion criteria were included in the study. The household transmission (SAR) of SARS-CoV-2 among household contacts was 58.8% (95% CI: 53.2-64.2) (Figure 1).

### 3.2. Characteristics of Index Patients with COVID-19

The median age of the index patients was 35 years (IQR 14 years). More index cases were adults than children (86% vs. 14%). Both sexes were equally affected. Index cases were detected from four different settings: mass screenings (*n* = 44), flu clinics (*n* = 24), hospital triage (*n* = 6), and contact tracing (*n* = 19) during the outbreak. Of the 93 cases, 72 (77.4%) patients were symptomatic. Symptomatic index cases had positive PCR at a median of 3 days of symptoms (IQR 5.5 days). All of these cases were mildly symptomatic (Table 1).

### 3.3. Characteristics of Household Contacts

The median household size was 4 (mean 4.3 ± 2.199) members. Compared to index cases, median age of contacts was younger, with 22 years (IQR 23 years). The median time from symptom onset of index cases to symptom onset in household contacts was approximately 3 days (IQR 2 days). Overall household contacts were mostly asymptomatic (*n* = 201, 65.7%) (Table 1).

### 3.4. Secondary Transmission of COVID-19

Viral transmission was observed in 69 of the 93 households (74.3%, 95% CI: 64.2-82.2). In 41 index case households, all contacts were infected while no secondary transmissions took place in 24 households. Secondary transmission was confirmed in 180 of the 306 household contacts (SAR 58.8%, 95% CI: 53.2-64.2). Seven contacts were isolated together with the index as dependents/caregivers out of which 2 contacts did not get infected even at the end of the 21-day isolation period. One household contact who had recently recovered from COVID-19 was reexposed to an index case and did not get reinfected. None of the cases were severe, and there were no mortalities reported.

### 3.5. Susceptibility of Household Contacts

Overall, children contacts had a marginally higher SAR compared to adult contacts (63.4% vs. 55.7%). On further age stratification, adults 45 to 59 years had a higher SAR compared to other age groups (69.6%). Almost all of the pediatric household cases (95.2%, 80/84) were secondary to an adult case, and 4.8% (4/84) were secondary to another child. Similarly, among adult contact cases, a majority (88.5%, 85/96) was infected by an adult index case, and only 11.5% (11/96) were secondary to pediatric index cases. When stratified by relationship to the index case, children (<18 years) of the index case (72/99, 72.7%) had the highest SAR followed by friends and neighbors (26/39, 66.7%) of the index case.

All but one (99%, *n* = 104) of the symptomatic contacts turned out to be infected (Table 2).

Asymptomatic infections are more common in children (40/84, 47.6% vs. 34/96, 35.4%), whereas adult contacts had more symptomatic infections (62/96, 64.6% vs. 44/84, 52.4%).

### 3.6. Transmissibility of Index Cases

Adult index cases had a higher transmission potential compared to children index cases (167/267, 62.5% vs. 13/39, 33.3%). Index cases who were asymptomatic had a lower transmission rate than symptomatic cases (38/73, 52.1% vs. 142/233, 60.9%).

The majority of the household contact cases (167/180, 92.8%) were infected after being exposed to vaccinated index cases. SAR among household contacts exposed to vaccinated index cases was higher to those contacts exposed to unvaccinated index cases (65% vs. 26.5%). When stratified for age, 76.5% (13/17) of the unvaccinated index cases were children <18 years (Table 2).

### 3.7. Statistical Analysis for Risk Factors of Infectivity

Age, sex, relationship, symptom status, comorbidity, vaccination status of contacts, and household size were analyzed as potential risk factors for SARs-CoV-2 transmission.  Table 3 shows the unadjusted prevalence ratios of the factors for infectivity. Further adjusted ratios were analyzed for near significant factors (Table 4).

Apart from age and vaccination status of the index case, none of the other factors were statistically significant. Prevalence ratio was 1.88 (95% CI 1.19, 2.95) times more among adult indexes compared to children. This difference was statistically significant, with a *p* value of 0.007. This significance was maintained when the prevalence ratios were further adjusted (aPR 1, 95% CI 1, 1.02; *p* = 0.01) (Table 4).

Similarly, a significant prevalence ratio of 0.41 (95% CI 0.25, 0.66) times more among those exposed to vaccinated index cases compared to those exposed to unvaccinated index cases was noted (*p* < 0.001) (Table 3).

## 4. Discussion

Our study findings of a high SAR for SARS-CoV-2 consolidated the known fact of SARS-CoV-2 infection rates being higher than the infection SARs of other coronaviruses such as SARS-CoV and Middle East Respiratory Syndrome-CoV [16, 17].

Among various transmission models, household transmission has been identified as major drivers for SAR [18]. Our overall estimated household SAR was considerably higher than the SAR estimated by previous systemic reviews and meta-analyses by Madewell et al. [11, 16] (16.6%, 18.9%) and Thompson et al. (21.1%) [19]. Household SARs in recent studies vary widely, with studies in Singapore 6% [15] and Rawanda 1.77% [20] showing the lowest incidence, while others reported rates closer to ours (53% and 60%) [21, 22].

Significant heterogeneity in transmission rates are dependent on the sociodemographic, environmental, and behavioral factors in study population [23]. The present study was conducted during an outbreak period where strict lockdown measures were implemented. Even though these measures reduced movement and interactions, it had also inadvertently bound families inside their homes. Longer and more frequent interactions within a closed environment among household members would increase the rates of household transmission of SARS-CoV-2. It is also known that social distancing and masking practices are difficult to achieve in practice inside the household.

Another factor could be based on the differences in testing, isolation, and follow-up protocols. Frequent testing increases the yield for identifying secondary infections and results in higher SARs [24]. Also, studies with longer follow-up (21 days or more) have reported higher SARs [22, 25]. With our rigorous follow-up period of 21 days and frequent testing protocol (minimum of 4 times) irrespective of symptoms, we believe that our estimates of SARs may be more representative of the true household infection rates.

However, the high SAR could also be associated with the lack of preexisting immunity against the virus [26], or it could be as a result of the high transmission potential of the Delta variant which was the predominant strain during the study period. SARS-CoV-2 Delta variant is known to be 1.7 times more transmissible compared to the Alpha variant in setting of household transmission [27].

### 4.1. Susceptibility

Most household studies across the world have reported significantly lower transmission in children than to adult contacts [28–30]. In contrast to these observations, we found that children contacts had higher transmission rates compared to adults. Children of the index cases had 1.3 times the prevalence of secondary SARS-CoV-2 infection compared to contacts of other relationships. Our findings were also corroborated by similar findings of higher SAR in children of index cases done in US setting (42%) [31]. The higher infection rates in children can be influenced by the household behavioral and environmental factors [32] such as the close contact of parents with their children at home and also during the quarantine period, resulting in longer sustained viral exposure.

We believe that as a result of our strict testing and predefined interval testing of contacts, irrespective of age or symptoms, it shows the true susceptibility of children in a household setting.

### 4.2. Transmissibility

The SAR was significantly higher in households with adult index cases, consistent with previous reports [33]. This suggests that infectivity generally increases with age which is supported by studies on viral shedding [34, 35]. On the other hand, lower SAR in households with children index cases could mean that children are less likely to transmit the infection and are unlikely to be main drivers of the pandemic as reported by previous studies [29, 33, 36, 37].

However, children could have been infrequently identified as the index case because of their asymptomatic infections or due to the lockdown measures limiting their movements [38].

Vaccine effectiveness against symptomatic disease and mortality has been corroborated in recent observational studies [39, 40]. Nevertheless, it may not prevent household transmission of the Delta variant, where exposure is known to be close and prolonged [41]. Even though we reported a higher SAR among household contacts exposed to vaccinated index cases compared to those exposed to unvaccinated index cases, there were no severe disease or mortality from the infection.

Recent evidences from a meta-analysis corroborate similar findings of lower transmission to household contacts from fully vaccinated index cases but not from partially vaccinated index cases [42]. This could also be true for us since all of the vaccinated individuals had received one dose prior to getting infected.

Additionally, the high SAR even after vaccination can be a result of postvaccination breakthrough infections. These cases are increasing exponentially worldwide, especially caused by the highly transmissible Delta variant, which was the predominant variant during our study period as well. Though current vaccines are proven to be protective, the variant has shown capabilities of evading the immune system leading to these breakthrough infections [43].

Furthermore, the short time since vaccination and the small sample size of index cases in our study should also be considered before we presume this as a lack of vaccine effectiveness.

### 4.3. Limitations

Our study is not without some limitations. Firstly, the index case may not always have been the primary case in the household. In addition, we identified only one individual as the index even in households where there was more than one positive case on the same day of testing based only on symptom onset. This could have led to some classification bias of index cases in the household, further affecting the risk factor analysis. Second, as a result of lockdown measures, predetermined mass screening timings could have delayed early inclusion and testing of eligible households, thereby further delaying correct identification of the transmission line. Additionally, only adults and children 5 years and older were included in the mass screenings. This could potentially explain the higher proportion of adult index cases. Third, we did not collect information about behavioral parameters that might have influenced transmission dynamics within households. Being a crowded city with compact housing, transmission between different households is highly possible and probable. Fourth, our household sample was small, which affected the strength of our risk factor analysis. For future studies, longer study duration could be considered for inclusion of significant number of households to improve the analysis. Finally, these findings are specific to the predominant variant at the time of the study and could change with emergence of new variants in the future.

### 4.4. Strengths

The strength of the study is its relatively long follow-up of all household contacts and higher frequency of testing irrespective of symptoms. The study also provides information on transmission dynamics for other cities or countries in Southeast Asia that have similar household settings with comparable sociodemographic, environmental, and behavioral factors.

## 5. Conclusions

Household transmission is likely a major driving force in human-to-human SARS-CoV-2 transmission, especially in situations like public health-mandated stay-at-home orders. The high secondary infection rate corroborates the findings of high transmissions in settings where there is sustained and prolonged contact. With frequent outbreaks of SARS-CoV-2 prompting lockdown measures, early contact tracing and case identification with subsequent case isolation from other household contacts remains a crucial step in preventing secondary transmission. Barring which, effective strategies must be explored to reduce transmission during home isolation. Vaccines remain effective in preventing severe disease and mortality. However, it may not prevent household transmission of highly transmissible strains.

## Figures and Tables

**Table 1:** Demographic characteristics of index cases and their household contacts in Phuentsholing, Bhutan.

Characteristics	Index case (*n* = 93)		Household contacts (*n* = 306)	
	*n*	%	*n*	%
Age				
<18 years	13	14.0	123	40.2
18-44 years	66	71.0	146	47.7
45-59 years	10	10.8	23	7.5
≥60 years	4	4.3	14	4.6
Sex				
Male	46	49.5	131	42.8
Female	47	50.5	175	57.2
High risk				
Yes	25	26.9	0	0.0
No	68	73.1	0	0.0
Clinical status				
Symptomatic	72	77.4	105	34.3
Asymptomatic	21	22.6	201	65.7
Symptom status				
Mild	72	100.0	102	97.1
Moderate	0	0.0	3	2.9
Household size				
≤3 members	40	43.0	—	—
4-6 members	42	45.2	—	—
≥7members	11	11.8	—	—
Comorbid				
Yes	7	7.5	17	5.6
No	86	92.5	289	94.4
Vaccination (≥18 years)				
Yes	76	95.0	178	97.3
No	4	5.0	5	2.7
Doses				
1 dose	76	100.0	179	100.0
2 doses	0	0.0	0	0.0

**Table 2:** Characteristics of secondary transmission of COVID-19 in households.

Characteristics	Household contacts (*n*)	Positive household contacts (*n*)	Secondary attack rate (%)	95% CI
*Characteristics of household contacts*				
Age				
<18 years	123	78	63.4	(54.5-71.5)
≥18 years	183	102	55.7	
18-44 years	146	78	53.4	(44.1-61.4)
45-59 years	23	16	69.6	(47.9-85)
≥60 years	14	8	57.1	(30.7-80.1)
Sex				
Male	131	78	59.5	(50.9-67.7)
Female	175	102	58.3	(50.8-65.4)
Relationship to the index case				
Spouse	47	26	55.3	(40.9-68.9)
Siblings	39	16	41	(26.7-57.1)
Child	99	72	72.7	(63.1-80.6)
Parents	28	15	53.6	(35.1-71.1)
Relatives (grandparents, cousins, others)	47	22	46.8	(33-61.1)
Friend/neighbor	39	26	66.7	(50.4-79.7)
Employee	7	3	42.9	(12.9-79.2)
Clinical status				
Symptomatic	105	104	99	(93.4-99.9)
Asymptomatic	201	76	37.8	(31.3-44.8)
Symptom status				
Mild	102	101	99	(93.2-99.9)
Moderate	3	3	100	—
Comorbid				
Yes	17	10	58.8	(34.5-79.5)
No	289	170	58.8	(53-64.4)
Vaccination				
Yes	179	99	55.3	(47.9-62.5)
No	127	81	63.8	(55-71.7)
Vaccination (≥18 years of age)				
Yes	178	98	55.1	(47.6-62.3)
No	5	4	80	(25.3-97.9)
Doses				
1 dose	179	99	55.3	(47.9-62.5)
Past COVID infection				
Yes	1	0	0	—
No	305	180	59	(53.4-64.4)
*Characteristics of index case*				
Age of index case				
<18 years	39	13	33.3	(20.3-49.6)
≥18 years	267	167	62.5	(56.6-68.2)
Index case sex				
Male	156	91	58.3	(50.4-65.9)
Female	150	89	59.3	(51.2-67)
Symptom status of index case				
Symptomatic index	233	142	60.9	(54.5-67)
Asymptomatic index	73	38	52.1	(40.6-63.3)
Vaccination status of index case				
Yes	257	167	65	(58.9-70.6)
No	49	13	26.5	(16-40.7)

**Table 3:** Unadjusted prevalence ratio of risk factors for infectivity of SARS-CoV-2.

Risk factors	*n*	uPR (95% CI)	*p* value^∗^
*Factors of household contacts*			
Age			
<18 years	123	1	
≥18 years	183	0.88 (0.73,1.06)	0.174
Sex			
Male	131	1	
Female	175	0.98 (0.81, 1.18)	0.825
Relationship			
Spouse	47	1	
Relatives	83	0.81 (0.57, 1.15)	0.229
Child	99	1.31 (0.99, 1.75)	0.059
Parents/grandparents	31	0.93 (0.61, 1.43)	0.75
Others (neighbors/friends/employers)	46	1.14 (0.81, 1.6)	0.45
Comorbidity			
Yes	17	1	
No	289	1 (0.66, 1.51)	1
Vaccination			
Yes	179	1	
No	127	1.15 (0.96, 1.39)	0.133
Household size (categorical)			
≤3 members	64	1	
4-6 members	155	1.07 (0.83, 1.39)	0.591
≥7 members	87	1.13 (0.86, 1.5)	0.37
Household size	306	(0.95, 1.02)	0.426
Age	306	1 (0.99, 1)	0.396
*Factors of index case*			
Age of index case			
<18 years	39	1	
≥18 years	267	1.88 (1.19, 2.95)	0.007
Index case sex			
Male	156	1	
Female	150	1.02 (0.84, 1.22)	0.859
Symptom status of index case			
Symptomatic index	233	1	
Asymptomatic index	73	0.85 (0.67, 1.09)	0.203
Vaccination status of index case			
Yes	257	1	
No	49	0.408 (0.25, 0.66)	<0.001

uPR: unadjusted prevalence ratio. 1: reference value. ^∗^*p* values were calculated using the generalized linear models for binomial family.

**Table 4:** Adjusted prevalence ratio for factors of infectivity.

Factors^∗^	*n*	aPR (95% CI)	*p* value
Age of index case	93	1 (1, 1.02)	0.01
Age of contact			
<18 years	123	1	
18-44 years	146	1.3 (0.66, 2.46)	0.476
45-59 years	23	1.5 (0.72, 3.33)	0.264
≥60 years	14	1.3 (0.58, 2.73)	0.557
Symptom status of index case			
Symptomatic	233	1	
Asymptomatic	73	0.8 (0.66, 1.04)	0.107
Relationship			
Spouse	47	1	
Relatives	83	0.9 (0.63, 1.3)	0.599
Child	99	1.3 (0.91, 1.94)	0.139
Parents/grandparents	31	1.3 (0.78, 2.01)	0.352
Others (neighbors/friends/employers)	46	1.3 (0.91, 1.82)	0.152
Vaccination (contacts)			
Yes	179	1	
No	127	1.4 (0.72, 2.58)	0.335

aPR: adjusted prevalence ratio. 1: reference value ^∗^The variables with *p* value ≤ 0.2 from unadjusted regression analysis were taken into consideration for this model.

## Data Availability

The datasets used and/or analyzed during the current study are available from the corresponding author on reasonable request.

## Supplementary Materials

Supplementary Materials Supplementary file 1: STROBE checklist (Supplementary 1).
